# Assessing admitted patients’ preparedness during mass casualty incidents at the hospital – a prospective study

**DOI:** 10.1186/s13584-025-00716-1

**Published:** 2025-10-07

**Authors:** Winston Wolf, Lia Schoenfeld, Maya Siman-Tov, Moran Bodas, Bosmat Hoch, Kholood Abu Hamad, Ronit Bar-Haim, Shachar Laks, Katia Dayan, Mordechai Shimonov, Adam Lee Goldstein

**Affiliations:** 1 Department of Disaster Medicine, School of Public Health, Faculty of Medical and Health Sciences, Tel Aviv University, Tel Aviv, Israel; 2 Department of General Surgery, Wolfson Medical Center, Ha-Lokhamim St 62, Holon, 5822012 Israel; 3 Department of Emergency and Disaster Management, School of Public Health, Faculty of Medical and Health Sciences, Tel Aviv University, Tel Aviv-Yafo, Israel; 4 Surgical Oncology & Endocrinology Unit, Department of General Surgery, Wolfson Medical Center, Holon, Israel; 5 Head of Surgery Division, Wolfson Medical Center, Holon, Israel

**Keywords:** Mass casualty incidents, Disaster planning, Hospitals

## Abstract

**Background:**

Hospitalized patients are inherently one of the most vulnerable populations. When the hospital is damaged, this population becomes even more at risk. Violence and natural disasters always have the potential to damage hospitals and other healthcare facilities, creating additional strain on an already catastrophic event. This study presents the preparedness of hospitalized patients during a mass casualty incident in order to understand their perspectives during a time of war in order to prepare, optimize, and to guide policy in order to maintain the highest level of care, efficiency, and safety.

**Methods:**

A descriptive cross-sectional prospective analysis was conducted for patients admitted during an active war.

**Results:**

Data from 103 patients were analyzed. 77% reported mobility limitations that significantly increased compared to their baseline home status. The majority did not know what to do in a disaster, only 20.4% felt able to take care of themselves in an emergency. 61.7% believed it was important to have CPR or stop-the-bleeding training. The major challenges were getting out of bed (56.9%), mobility help (41.2%), pain, and attachment to IVs/drains (both 25.5%). Older patients, non-Jewish individuals, religious patients, and those without an academic degree were all significantly at higher risk for having less knowledge about what to do if there was a mass casualty incident.

**Conclusions:**

There is a clear need to better understand the patient perspective, as reflected in specific points identified in this study, in order to optimize hospital preparedness when the hospital itself is a disaster victim.

## Introduction

The occurrence of Mass Casualty Incidents (MCI) continues to increase in every corner of the world [1]. These incidents may be violence related (war, mass shootings), natural disasters (earthquakes, tsunami), or accidents (motor vehicle, infrastructure breakdown). MIC (of both human and natural origins) are indifferent to the socioeconomic status of a country, and therefore are a universal public health problem that must be prepared for appropriately in order to best organize the chaos, and help as many salvageable victims as possible [2–4]. No matter what the event is resulting in a surge of those injured, or the size of the health facility, the community and healthcare providers must have established protocols and conduct consistent simulation training in order to best be prepared and adequately treat the maximum number of victims.

A significant part of a hospital team’s preparation, especially during times of war, is preparing for the physical structure of the hospital being the ‘disaster victim’, an in-hospital MCI (IH-MCI) [5, 6]. This is unfortunately a well documented occurrence in the region in both past and current events [7, 8]. In the dire event that the hospital is damaged, and/or unsafe for the patients and staff, together with a surge of new patients, creates a catastrophic situation that makes an already challenging situation exponentially more difficult. Not only does the focus shift from doing the most for the greatest number of victims, but to also trying to keep currently hospitalized patients alive, safe from danger, and/or assessing the need to transfer them. The inability to take care of current or new patients is often due to loss (or damage) to certain aspects of the hospital’s infrastructure such as oxygen, water, and electricity [9, 10]. This is in conjunction with possibly injured hospital staff members, and/or the additional danger to staff members trying to treat, access, and/or mobilize hospitalized patients at risk.

Research has examined the healthcare staff’s feelings towards preparedness [11], and in retrospect after experiencing a MCI [12], yet there is no data examining how knowledgeable or prepared an in-patient population is towards an IH-MCI. In-patient populations are inherently one of the most vulnerable populations in any community and highly dependent on others. This susceptible population is sick, elderly, possibly post-operative, limited or with no mobility, or intubated and sedated. Therefore when a hospital is at risk, it is important that the inpatient population (together with the local staff) are prepared and understands protocols and necessary precautions if there was to be a IH-MCI. For the hospital administrating and medical teams managing the IH-MCI, it is also vital to understand the limitations and needs of their inpatient populations. For policy makers on all levels, this knowledge will optimize response and preparedness, allowing for more efficient care for the maximum number of victims. There are currently no studies examining this crucial aspect of patient care during a IH-MCI. During an on-going war in our region, we aim to better understand the current knowledge and limitations for a surgical in-patient population regarding IH-MCI in the event that the hospital was damaged.

## Methods

A descriptive cross-sectional analysis was conducted among patients admitted to the general surgery department at a single hospital during an on-going war over a two month period. (March and April 2024). A sample size of 100 responses was collected to represent the entire types of patients admitted to this department in this time period. Using Google Forms, a questionnaire was developed in the local language. Every admitted patient was both given a short sheet describing the study. Volunteers went around explaining the purpose of the study and obtained consent from the patients. If the patient was more comfortable in another language, then their native language was used for explanation and also, if they decided to proceed, to conduct the survey with the help of a volunteer fluent in their language. The study was accessed via a QR code and done on the patient’s private cell phone, or on a volunteer’s phone if the patient did not own a smartphone. Excluded were intubated patients, under the age of 18, with either acute delirium, or known dementia. Patients who spoke a language that we did not have a native speaker for were also excluded. The hospital’s internal review board approved of this study in compliance with the ethical standards of the Declaration of Helsinki.

The questionnaire consisted of both qualitative and quantitative survey questions. Quantitative data was obtained using a Likert Scale. Qualitative data was gathered through both open-ended and closed-ended questions. Statistical analysis was conducted with SPSS Statistical Software (version 29). Descriptive statistics were used to describe patients characteristics. A McNemar-Bowker Test was used to compare differences between baseline mobility at home verses during admission. In order to identify target groups for low level of knowledge regarding what to do in the event of an MCI at the hospital we divided the independent variable into three categories: Low level of knowledge (answer ‘1’), Moderate level of knowledge (answer ‘2’), and High level of knowledge (answer ‘3–5’). One-Way ANOVA and chi-square analysis was used to identify different levels of knowledge among different demographic characteristics (Age, Gender, religious, education and income). A p-value of < 0.05 was considered statistically significant.

## Results

### Demographics

A total of 103 surveys were analyzed, representing 103 admitted patients to the general surgery division. The sociodemographic characteristics of the patients are described in Table 1. The sample consisted of 48 males (46.6%) and 53 females (51.5%), with 2 (1.9%) not identifying with a gender. The average age was 59.13 years old (SD +/- 12.91 years, range 22 to 85). The religion the participants identified by consisted of 57.4% Jewish, 28.7% Muslim, 13.9% Christian, and 1.9% not answering this question. Concerning current family status, the majority were in a relationship without children (46.6%), followed by a relationship with children (34%), no relationship with children (13.6%), and not in a relationship and without children (3.9%). Only 13.9% of our cohort lived alone, and the remaining either lived with their children, a spouse, parents, or both partner and children (43.6%, 5.8%, 2%, and 33.7% respectively). 5% had a master’s degree or higher, 25% had a bachelors degree, 24% had some or all of highschool education, and 46% had a diploma from a trade school/course. The studied population described their income as average (38.6%), followed by low (31.7%), high (15.8%), very low (12.9%), and very high (1%). When asked if they had previous medical training, (such as basic life support, CPR, and tourniquet placement), 88 patients replied ‘no’ (85.4%), 10 replied ‘yes’ (9.7%), and 4 were ‘not sure’ (3.9%).

**Table 1:** Demographics

**Age (yr)** Mean +/- SD (Range)	59.13 ± 12.910 (22–85)	
**Gender n(%)**	Male	48 (46.6)
	Female	53 (51.5)
	Not Identifying with Gender	2 (1.9)
**Religion n(%)**	Jewish	58 (56.3)
	Muslim	29 (28.2)
	Christian	14 (13.6)
	No Response	2 (1.9)
**Family status n(%)**	In a relationship without children	48 (46.6)
	In a relationship with children	35 (34.0)
	Not in a relationship with children	14 (13.6)
	Not in a relationship without children	4 (3.9)
**Level of education n(%)**	Diploma (trade school)	47 (46)
	High School or below	25 (24)
	BA	26 (25)
	MA or higher	5 (5)
**Income level n(%)**	Very low	13 (12.9)
	Low	33 (31.7)
	Average	40 (38.6)
	High	16 (15.8)
	Very high	1 (1)
**Household composition n(%)**	Alone	14(13.9)
	With a spouse and children	35 (33.7)
	Single with children	45 (43.6)
	With partners	6 (5.8)
	With parents	2 (2)
	No response	1 (1)
**Baseline mobility limitations n(%)**	None	29 (27.5)
	Slight - rely on some help, but can move around independently	32 (31.4)
	Moderate - rely on lots of help, including with movement	37 (36.2)
	Severe - completely dependent on help	5 (4.9)
**Previous medical training n(%)**	Yes	10 (9.7)
	No	88 (85.4)
	Not sure	4 (3.9)

### Surgery characteristics

Within the patients surveyed, 26 underwent an operation during their current hospitalization (25.2%), and 76 patients did not (73.8%). Of those who had surgery the median time from surgery was 1 day, and range of 0 days to over 60 days.

### Mobility status

**Fig. 1:**
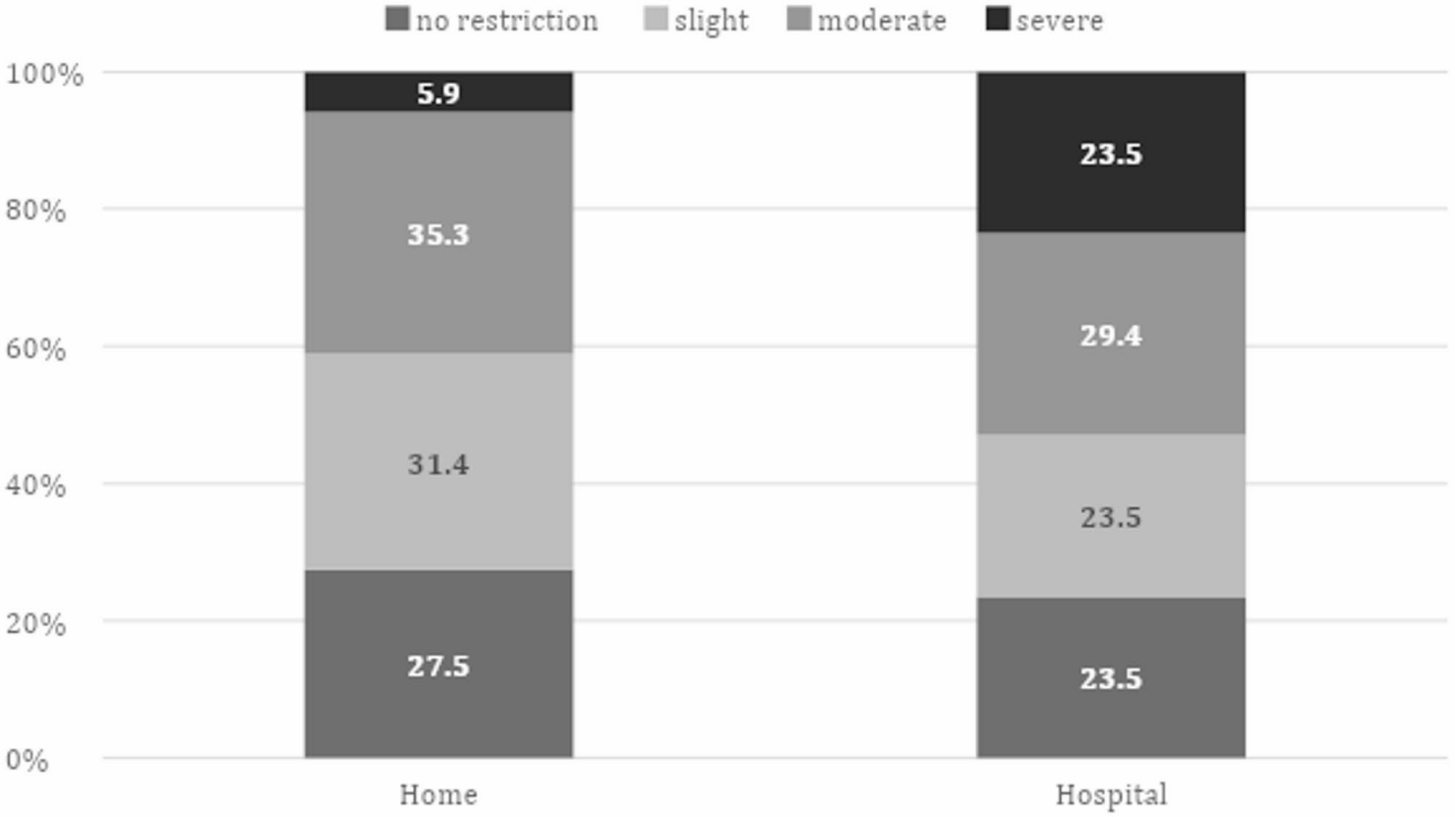
Differences Between Baseline Mobility at Home and During Hospitalization McNemar-Bowker Test = 15.30 (df = 6) *p* = 0.018

Mobility status before and during hospitalization presented in Fig. 1. Participants’ home baseline mobility status varied, with the majority stating that they had moderate mobility limitations (36.2%). This was followed by 31.4% declaring slight mobility limitations, 27.5% with no limitations, and 4.9% with severe mobility limitations. Concerning the cohort’s current mobility status during the hospitalization, 24 (23.3%) reported no limitations, 42 (40.8%) had slight limitations but could still get up and walk around independently, 30 (29.1%) had moderate limitations and felt they needed help moving around, and 6 (5.8%) said that they were completely dependent on others for any movement. There was a significant decrease between the mobility status before and during hospitalization. The median score before hospitalization was a 2 (slight mobility limitations but can move around the house independently), versus 3 during hospitalization (moderate mobility limitations and sometimes I need assistance or mobility aids). This difference was significant with a p-value of 0.018.

### Current knowledge of MCI & what to do in the event of an IH-MCI

The majority of patients did not know of the phrase ‘mass casualty incident’ (*n* = 77, 74.8%). If they were familiar with MCI, the most common places they received information and/or training about an MCI were during their time in military service (*n* = 48, 47.1%), from friends and/or family (*n* = 36, 35.3%), their workplace (*n* = 11, 10.8%), or social media/television ((*n* = 11, 10.8%). Twenty-three patients (22.5%) said they have never received information or training, and only 1% (*n* = 1) said that they received training and/or information via a hospital and/or health facility.

If they indicated that they did not know what an MCI was, it was explained to them in a single sentence: “The number of injured at an incident that overwhelms the hospital” [13]. After this explanation, they were asked if they have ever been involved in an MCI, 42.2% (*n* = 43) stated they have been injured to some extent in a past event, 38.2% (*n* = 39) have observed an event, 25.5% (*n* = 26) were never involved in an MCI, at 3.9% (*n* = 4) were actively working in a MCI as part of the emergency services in the field (police, ambulance, fire, or military). In respect to knowledge or training the cohort felt important in the event they were involved in an MCI, the majority replied knowledge of who to contact for help (64.7%, *n* = 66), followed by knowledge of basic CPR (36.3%, *n* = 37), knowledge of basic bleeding control (25.5%, *n* = 26), and knowledge of where to go for safe shelter (1%, *n* = 1). Our cohort’s knowledge regarding an MCI is outlined in Table 2.

**Table 2:** Knowledge & preparedness of MCI

**Familiar with phrase ‘mass casualty incident’ n(%)**	yes	25 (24.3)
	No	77 (74.8)
	No response	1 (1)
**Have you been involved in a MCI before? n(%)**	Have been injured in an MCI	43 (42.2)
	Have observed an MCI	39 (38.2)
	Never involved in an MCI	26 (25.5)
	Worked as emergency service member during an MCI	4 (3.9)
**What training/knowledge is important to know in case of an MCI? n(%)**	Who to contact for help	66 (64.7)
	Basic CPR	37 (36.3)
	Basic bleeding control	26 (25.5)
	Where to go for safety	1 (1)
**What way would you like to receive more information about preparedness/awareness of hospital protocols during an MCI? n(%)**	Written/Pamphlet	43 (41.7)
	Verbal explanation from staff	43 (41.7)
	Not interested in receiving	9 (8.7)
	Via the telephone/internet	2 (1.9)
**Do you know what to do in the event of an MCI at the hospital? n(%)**	1 (not know at all what to do)	22 (21.6)
	2	62 (60.8)
	3	8 (7.8)
	4	8 (7.8)
	5 (knowing exactly what to do)	2 (2)
**Do you feel independent & able to take care of yourself during this hospitalization if there was an MCI? n(%)**	No	77 (74.8)
	Yes	21 (20.4)
**The major challenges you would face during an MCI? n(%)**	Difficulty to get out of bed and/or lower the bed rail	58 (56.9)
	Dependent on help for mobility	42 (41.2)
	Attached to drains and/or IVs	26 (25.5)
	No limitations	1 (1)

When identifying at-risk groups for lacking knowledge of what to do in the event of an IH-MCI, younger patients, those identifying as Jewish, non-religious, and those with an academic degree were all significantly more likely to have higher knowledge levels compared to older patients, non-Jewish individuals, religious individuals, and those without an academic degree (*p* < 0.001, *p* = 0.023, *p* = 0.009, and *p* = 0.003 respectively). Demographic characteristics associated with the level of knowledge of what to do in the event of an IH-MCI is outlined in Table 3.

**Table 3:** Demographic characteristics associated with the level of knowledge in the event of an IH-MCI

	Level of knowledge of what to do in the event of an IH-MCI			*p*-value*
	Low level (1) *N* = 22	Moderate level (2) *N* = 62	High level (3–5) *N* = 18	
**Age (yr)**	67.3 ± 11.1	58.6 ± 10.9	50.6 ± 15.7	< 0.001
**Gender**				
Male	18.8%	62.5%	18.8%	0.884
Female	22.6%	60.4%	17.0%	
**Religion**				
Jews	27.6%	50.0%	22.4%	0.023
None Jews	11.6%	76.7%	11.6%	
**Level of religiosity**				
Secular	33.3%	42.9%	23.8%	0.009
Religious	13.6%	72.9%	13.6%	
**Income**				
Low	22.2%	68.9%	8.9%	0.296
Moderate	23.1%	51.3%	25.6%	
High	17.6%	58.8%	23.5%	
**Education**				
High school or below	45.8%	37.5%	16.7%	0.003
Diploma (trade school)	19.6%	69.6%	10.9%	
Academic	6.7%	63.3%	30.0%	

*P-value based on One-Way ANOVA for continuous variables (Age) or Chi square analysis for categorical variables (Gender. Religion, Level of religiosity, Income and Education)

### IH-MCI preparedness

For the question, in what way would you like to receive more information about the preparedness/awareness of hospital protocols during a mass casualty event? 41.7% (*n* = 43) of the participants prefer written information such as a pamphlet, 41.7% (*n* = 43) of the participants prefer a verbal explanation from a staff member, 8.7% (*n* = 9) of the patients were not at all interested in receiving information, and 1.9% (*n* = 2) prefer receiving information over the phone/internet.

When asked if they know what to do in the event of an emergency (IH-MCI) at the hospital, on a scale of 1 to 5 (1 not knowing at all what to do, 5 knowing exactly what to do), 60.8% (*n* = 62) responded with a 2, 21.6% (*n* = 22) respond with 1, 7.8% (*n* = 8) responded with a 3 and 4, and only 2% (*n* = 2) respond with a 5. Only 20.4% (*n* = 21) felt independent and able to take care of themselves in an emergency situation, and 74.8% (*n* = 77) did not feel able to take care of themselves during a IH-MCI. The major challenges faced according to the patient population were, in descending order, difficulty to get out of the bed/lowering bed rail (56.9%, *n* = 58), dependence on help for mobility (41.2%, *n* = 42), pain and attachment to drains and/or IV’s (both 25.5%, *n* = 26), and only 1% (*n* = 1) reporting no limitations. Our cohort’s preparedness regarding an IH-MCI is outlined in Table 2.

## Discussion

In the catastrophic event of a damaged hospital, taking care of the in-patient population is an extreme challenge that must be understood and optimally prepared for by all parties involved. The influx of new patients, the added resources needed to treat injured in-patients, and the possible additional risk placed on the medical staff meeting these needs are all complex components that must be taken into consideration in order to adequately treat the maximum number of victims [14]. Despite the possibility of a IH-MCI occurring at a healthcare facility remains low, there are specific geo-political locations that will have a higher risk of either natural or violent (or both) events. During wars [15], civil unrest [16], and higher risk areas for non-seasonal natural disasters (earthquakes, tsunami) [17, 18], and seasonal natural disasters (hurricanes, tornadoes) [19], entire communities are at heightened risk. In these locations, hospitalized populations are one of the most vulnerable that must be accounted for, adequately educated, and maximally empowered in order to decrease morbidity, mortality, and overall burden on the hospital and staff. Policy makers, from the local hospital administration to the overseeing government agencies, need to understand the needs outlined in this study in order to ensure that adequate policy is established to meet the needs of this at-risk population. Optimal preparedness and care for hospitalized patients during an in-hospital disaster comes from routine practice, simulation/drills, and education of the hospital’s staff. Yet, this study may help to better prepare and focus on aspects of these training on practical issues that the patients are facing. Our results, and our ‘Key Points’ shown below are relevant and adaptable to any geographic and socioeconomic setting, with slight modifications depending on the specific details of the hospital ward and patient population.

This study shows a clear lack of knowledge, preparedness, and heightened feeling of dependence/decreased mobility in our hospitalized cohort. We also identify at-risk groups within the cohort who are less knowledgeable about what to do in the occurrence of an IH-MCI. Despite decades of violent turmoil in the region, encompassing varying degrees of intensity, and the frequent occurrence of MCIs (both conflict- and non-conflict-related) [20, 21], there remained a notable lack of familiarity with the term “mass casualty incident.” This did not correlate with the surprisingly high number of responses claiming to have been injured or witnessed an MCI. This inconsistency most likely represents a misunderstanding of the questions, or a lack of formal training and definitions provided to the general public regarding an MCI. Hence, many citizens may have been involved or witnessed without understanding the overall situation. We also could not find any literature with population-level surveys asking the general public whether they have ever been injured in an MCI. Our results are in line with ‘The Victimization Model’ coined by Turkish researchers after a major earthquake, and Israel’s own experience through a violent history, where under constant threat the local population restores to denial as an adaptive coping mechanism [10, 22]. Hence avoiding the thought or acknowledgement of MCI’s, and any related preventive training. Once this is acknowledged, important training may be conducted catered to the psychosocial factors of this phenomenon allowing optimal results and comprehension without increasing personal traumas.

Adequate understanding by the public about the unique needs of an IH-MCI may improve a common citizen’s (or patients) response, and immediate actions, in order to help the chaotic situation instead of adding to the disarray [23]. The results of this study may also be useful information for non-MCI situations, where there are no (or not many) injured, yet part of the hospital has been damaged and is a risk for the hospitalized patients. For inpatient populations, better understanding the acute needs of the situation, may stimulate more mobile patients to ‘step-up’ and help other patients needing assistance. Patients will need to know, just like anyone else (the hospital administration, the staff, families), what to do in the immediate situation without having to depend on the leadership of hospital/ward staff who may be overburdened or potentially injured themselves.

A majority of patients identified either CPR or bleeding control as important knowledge to know in case of involvement in an IH-MCI. It would be unrealistic for admitted patients to undergo formal CPR (basic life-support) or stop-the-bleeding training. Yet, this may be basic training information that could be made available to all hospitalized patients and their families through a pamphlet or short internet video from one’s phone/tablet. Examples of relevant information for the patients can be airway clearance, c-spine precaution, placement of an airway device, putting pressure on bleeding, and tourniquet placement [23]. In high-risk geographic areas, and/or during war times, a placement of a ‘civilian kit’ in hospital wards might help save lives. These kits, much like civilian defibrillators and resuscitation trolleys on hospital wards [24], may include basic airway and stop-the-bleeding equipment, and be readily accessible for both patient and staff use in the event that the hospital is damaged. For example, teaching highschool students ‘light’ search-and-rescue skills in an earthquake-prone region showed improved resilience, self-efficacy, and knowledge [25, 26]. We believe that basic training will help fellow patients and families ‘step-up’, and feel more empowered and confident to assist during the most challenging of times. The fact that CPR was identified is an important point because on the one hand CPR should not be attempted during an MCI (or IH-MCI) due to the overwhelming of available resources. Yet, on the other hand, non-medical personnel should not be making this decision. Therefore further expert discussion needs to address this and establish appropriate protocols.

The identification of these major challenges seen through the eyes of the patients are important points to address for any government, hospital administration, and ward. Especially since the majority of patients did not feel independent at the time of being questioned.

### Policy implications & recommendations

On the national Level, disaster response leaders (from a Ministry of Health or other overseeing government body) need to identify at-risk hospitals, and if possible allocate additional resources, require specific trainings, and develop protocols that would better prepare the hospital to maximize the safety and health of the vulnerable in-patient population. This obviously may vary with season or violent conflict in the region, yet we recommend that there are set standards and plans to be activated as necessary, along with a defined leadership structure to initiate these protocols as needed. This is relevant in both high and low income countries, where the ‘national plans’ for better protecting in-patient populations can be adjusted and tailored to the abilities and resources available. Examples of possible equipment needs are mentioned above such as the ‘civilian kits’ available in the departments.

For the hospital administration, they must also recognize when their patient population is at an increased risk, know who to reach out to for government assistance, and appoint personnel to lead in-hospital disaster preparedness, monitor the specific actions taken by the hospital wards, and implement protocols and trainings. The hospital leadership, together with the individual departments need to work together to optimize the conditions on the ward regarding disaster preparedness, and maintain a certain level of understanding, training, and education of the department’s staff. Based on the findings in this study, simple policy and a few minutes of education can enable a patient to feel more secure and independent. For example, simple practical instruction and practice opening the bed rail can significantly improve the patient’s confidence and self-sufficiency during a disaster. By understanding the challenges shown in our data, hospital wards at times of increased risk can also use ‘buddy systems’ when placing patients in rooms together. For instance, a young healthy male undergoing a simple appendectomy, can be placed next to an elderly man who is dependent on help. Simple instructions directed to the patient and/or family on how to close or disconnect an IV line might also be useful during a disaster situation.

**Fig. 2:**
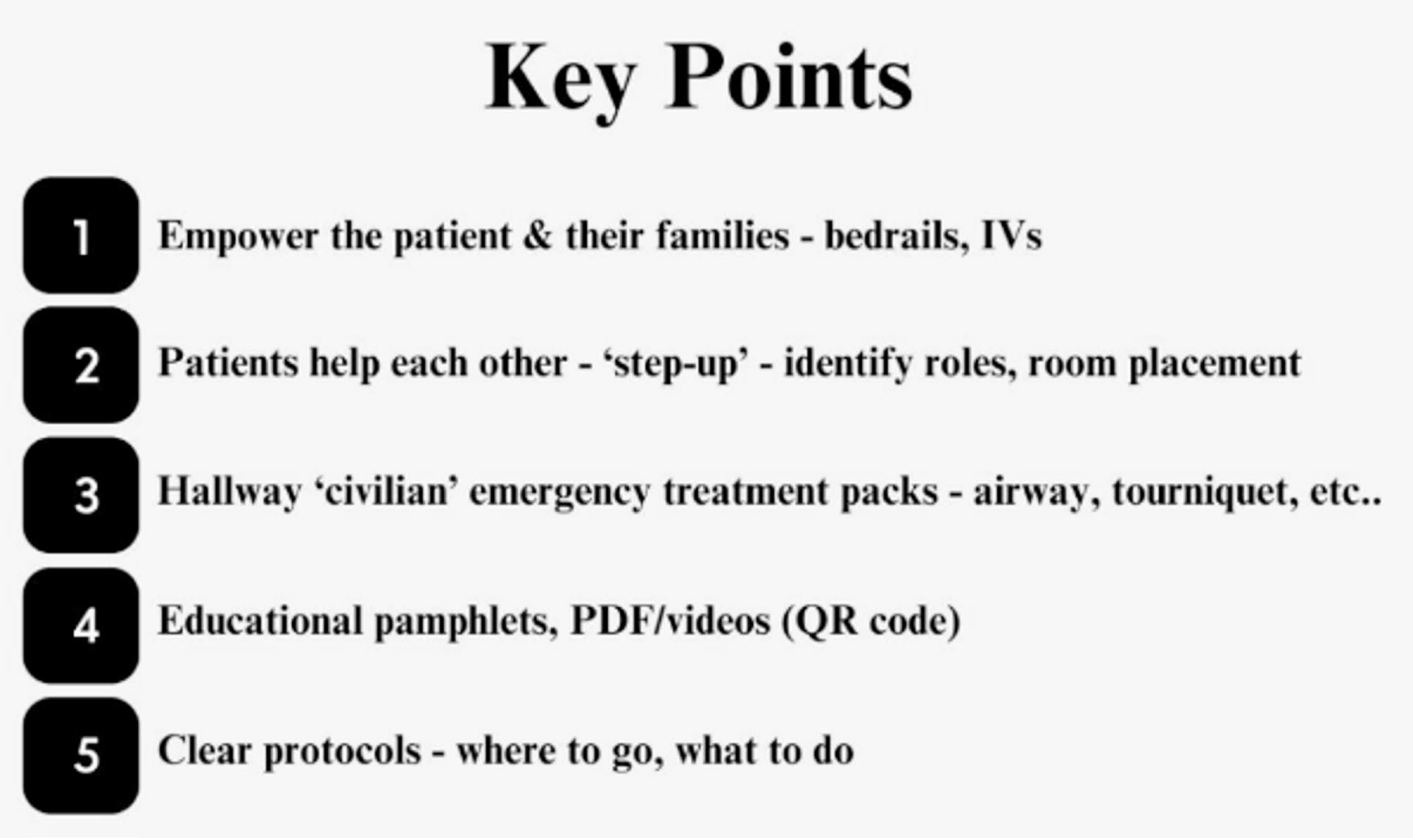
Key points

Figure 2 summarizes the major policy key points on how to improve readiness for, and the patient’s perspective of, a IH-MCI.

### Limitations

This study is limited by being conducted in one country, in a single ward at one hospital. We believe that general surgical/trauma patients are inherently some of the most vulnerable, though different departments/wards might have unique needs to their patient population. Due to the fact that these are surgical-ward patients, they might have a more significant limitation in mobility. There is also a selection bias due to the fact that not everyone who was approached wanted to take the survey. Those without ‘smartphones’ were given the opportunity to complete the questionnaire with a volunteer, yet this might have also hindered their motivation/desire to complete the questionnaire. Intubated and sedated patients were excluded from this study, which is one of the most vulnerable and difficult-to-manage patient groups during an in-hospital disaster. Further studies need to examine how to best manage this specific group during these types of situations.

## Conclusion

IH-MCI is a catastrophic event that calls for extreme actions both by the hospital and the inpatient population. Despite the limitations, our results show a significant need for improvement and patient directed education during unique, and unfortunately not uncommon, circumstances. Larger studies conducted in different geopolitical contexts, and a variety of hospital departments, can further identify key points that will save lives when the hospital is a victim. There is a clear need to improve hospitalized patients’ education and preparedness for the event that the hospital is a ‘disaster victim’. Here we have described higher at-risk populations’ perception during a war, together with methods to improve patients’ empowerment and care during an IH-MCI. For local and regional policy-makers and hospital administrators, these results demonstrate practical needs of this population, and suggest actions to improve the efficacy of disaster response and meet these needs. This will not only save lives of the vulnerable hospitalized population, it will also improve the ability for the hospital and its staff to take better care of more patients.

## Data Availability

No datasets were generated or analysed during the current study.
